# Energy balance at the organism and cellular level: effects of biguanides

**DOI:** 10.1186/2049-3002-2-S1-O26

**Published:** 2014-05-28

**Authors:** Michael Pollak

**Affiliations:** 1 McGill University and Jewish General Hospital, Quebec H3T 1E2, Canada

## 

Metformin, an inhibitor of OXPHOS, is widely used for treatment of type II diabetes (T2D). A key site of action in diabetes treatment is liver, where the drug achieves a relatively high concentration following oral administration, leading to inhibition of gluconeogenesis and reduction of the hyperglycemia and hyperinsulinemia of T2D. As high levels of insulin have been associated with poor prognosis of prostate, breast, and colon cancer and as early retrospective pharmaco-epidemiologic studies suggested reduced cancer burden among diabetics treated with metformin relative to other diabetes treatments, the hypothesis that metformin or other biguanides could be useful in cancer prevention or treatment has received considerable attention. However, it is not clear if metformin at conventional anti-diabetic doses administered to non-diabetic subjects has effects of sufficient magnitude on levels of insulin or other candidate mediators to influence cancer biology. Drugs used for androgen deprivation in prostate cancer treatment and most PI3K inhibitors provide examples of therapies that induce hyperinsulinemia which may attenuate therapeutic benefit, justifying study of combinations of each of these with biguanides. The separate hypothesis that biguanides can directly influence cancer cell metabolism is attractive, as this mechanism has been shown to operate in many laboratory models - yet there is uncertainty if metformin administered at conventional anti diabetic doses achieves sufficient concentrations in extra-hepatic tissues or cancers to perturb their cellular energy metabolism. Therefore, studies of maximally tolerated doses of biguanides with more favourable pharmacokinetic properties than metformin may be necessary if the “direct action” hypothesis is to be clinically tested. Recent work is identifying contexts where ‘direct’ actions of biguanides as inhibitors of OXPHOS may be of particular therapeutic benefit. For example, there is evidence that tumours using glutamine as a carbon source are relatively sensitive to biguanides, that high ambient glucose levels are associated with reduced sensitivity, and that serine deficiency enhances the anti-proliferative actions of biguanides.

